# Preparation of *Thymus vulgaris* (L.) essential oil nanoemulsion and its chitosan encapsulation for controlling mosquito vectors

**DOI:** 10.1038/s41598-022-07676-5

**Published:** 2022-03-14

**Authors:** Parisa Gupta, Shabad Preet, Ananya, Navneet Singh

**Affiliations:** Present Address: Department of Zoology, Faculty of Science, Dayalbagh Educational Institute, Dayalbagh, Agra, U.P. 282005 India

**Keywords:** Zoology, Diseases

## Abstract

Here, we report a novel comparative assessment of preparation and characterization of thyme oil nanoemulsion and its chitosan encapsulation using high energy approach for the management of three major mosquito species viz*., Anopheles stephensi* (Liston,1901), *Aedes aegypti* (Linn., 1762) and *Culex tritaeniorhynchus* (Giles, 1901). The synthesized formulations were analysed for thermodynamic stability, indicating 1:0.5 (oil: surfactant) ratio to be the most stable of thyme oil nanoemulsion while 1:1 (nanoemulsion: chitosan solution) ratio of its chitosan encapsulation. These were further characterized by dynamic light scattering and transmission electron microscopy which revealed the size and morphology of the droplets which measured 52.18 ± 4.53 nm for thyme oil nanoemulsion and 50.18 ± 2.32 nm for its chitosan encapsulation. All the droplets were well dispersed with distinct flower-shaped nanoemulsion and somewhat mitochondria like chitosan encapsulation. In-vitro release study of thyme essential oil from its nanoemulsion and chitosan encapsulation showed that 91.68% and 73.41% of the total oil concentration in water was released respectively to the environment after 48 h clearly depicting controlled release in the encapsulation. Assessment of insecticidal potential against selected mosquito species revealed that both the nanoemulsion and its chitosan encapsulation were effective on the major mosquito species. Maximum activity of thyme oil nanoemulsion was noticed against *C. tritaeniorhynchus* (LC_50_—22.58 ppm) after 24 h of exposure while it was observed that its chitosan encapsulation was most effective on *A. stephensi* (LC_50_—18.88 ppm) after 24 h of exposure. Consistent morphological alterations could be noticed in the larvae of mosquito species. Hence, these nanoemulsions and encapsulations could be further tested for their applications against other insect pests in agriculture.

## Introduction

Mosquitoes are considered as insect menace worldwide due to their role in the transmission of deadly diseases in humans. According to the latest World Malaria Report*,* released in January 2020 by the World Health Organization (WHO), there were 228 million cases of malaria in 2018 and an estimated number of deaths due to malaria stood at 405,000^1^. From India alone as per the records of National Vector Borne Diseases Control Programme (NVBDCP), in 2018, 429,928 cases of Malaria were reported with 96 deaths^2^. *Aedes aegypti* is the major species known for transmitting several arboviral diseases such as dengue, chikungunya and recently emerged for Zika virus. According to a recent report estimating 3.9 billion people, in 128 countries are at the high risk of dengue infection^3^. According to the WHO, almost 3 billion people in 24 countries of South-East Asia are at the risk of Japanese encephalitis virus transmission through the bite of *Culex tritaeniorhynchus*^4^.

The burden of these drastic diseases and the grave picture as painted by the data above calls out for immediate and far-reaching control methods against mosquito vectors. Conventional ways to tackle this problem consist of the use of chemical methods like synthetic pyrethroid pesticides viz*.* permethrin, resmethrin, sumethrin and malathion as adulticides and methoprene and pyriproxyfen as larvicides. Though highly efficacious, pesticide applications are facing a threat due to the development of resistance to chemical insecticides in mosquitoes and may result in a bounce of their vectorial ability^5^. Thus, attention needs to be turned towards low negative impact methods like the use of natural plant extracts, herbal components and essential oils to act as bio- insecticides and larvicides.

Thyme oil is a complex of essential oils extracted from fresh or partly dried flowering tops and leaves of *Thymus vulgaris* (L.) by the process of steam distillation. Usually essential oils present monoterpenes and sesquiterpenes and in some cases phenylpropanoids in their composition^6^. Thyme oil is one of the utmost commercial oils used worldwide and known for its various properties like food preservative, antioxidant, antibacterial, and antifungal^7^. Thyme oil is highly effective for larvicidal activity, strong anti-microbial properties, anti inflammatory and acaricidal^8,9^. However, phyto-components of thyme oil are volatile and release quickly to the environment therefore, for enhancing the efficacy and for the control release, the formulation has been encapsulated^10^.

Another component of interest in the delivery of chemicals is nanotechnology. The word “nano” is derived from Latin, signifying “dwarf” and mathematically is the billionth part of a meter. Nanotechnology is the design and synthesis of materials at a nano scale and use of their properties at an atomic or molecular level. But not all is positive with the use of insecticidal nanoparticles, various negative impacts of nanoparticles have also been observed like their accumulation in the organs of several animals including humans leading to nano toxicity^11^.

A recent trend of using herbal products in nano form is the fabrication of nanoemulsions for their biocidal activities owing to their relatively non-toxic and non-irritant nature. Since last decade, nanoemulsions were the topic of interest for the researchers working in the field of nanotechnology. Nanoemulsions synthesized by numerous methods from different plants like *Azadirachta indica* (A.Juss., 1830), *Pterodon emarginatus* (Vogel) exhibited larvicidal activity^12,13^. Repellent properties of essential oil based nanoemulsions were also recoded^14–16^. Other properties like antioxidant, insecticidal and antibacterial were also studied^17–20^. Nanoemulsion encapsulation comes into play when larvicidal potential and viability of essential oils is highly compromised due to their hydrophobic and volatile nature. Encapsulation is defined as a technology for packaging small solid particles, liquid droplets or gas molecules in a form that can release the contents at a controlled rate under specific conditions^21^. Essensial oil based nanocapsule are also considered potent as larvicidal activities^22^. Nano encapsulation of essential oils improves water dispersion and prevents their degradation. Encapsulation methods may increase the efficacy period while assuring the gradual and controlled release of the active substances of the essential oil^14,23^. Encapsulating materials namely, chitosan, potato starch, gelatin are derived from natural sources are the best option for encapsulation since they would have minimal hazardous effects and would be bio degradable as well^24^. Chitosan is a naturally occurring linear polysaccharide of glucosamine and N-acetylglucosamine that comes from the deacetylation of chitin, which is also present in the skin of mosquito larvae. Chitosan nanoparticles have attracted increased attention in the past decade due to their unique properties including non-toxicity, biocompatibility and biodegradation^25^.

Hence, in the present study, thyme oil nanoemulsion and its chitosan encapsulation were prepared using high energy ultrasonication approach and characterized for thermodynamic stability and release profile. Further, their application for enhancing larvicidal efficacy in controlling three major mosquito vectors viz*. Aedes*, *Anopheles* and *Culex* species.

## Results

### Gas chromatography and mass spectrometry (GC–MS)

**Figure 1:**
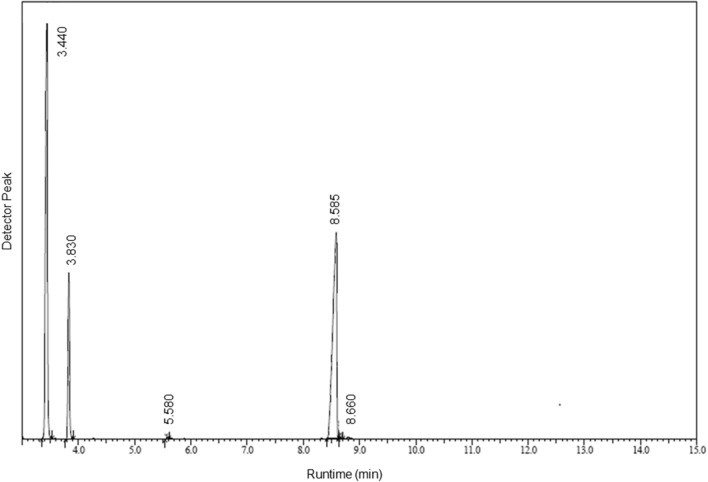
Phyto-compounds detected in thyme essential oil using GC–MS.

GC–MS analysis of thyme essential oils was presented in Table 1, Fig. 1. Five major compounds were found in the oil which were classified as monoterpenes and phenol. 1,3,8-p-Menthatriene is classified as monoterpene and was present in maximum concentration (45.58%) while Phenol, 2-ethyl-4,5-dimethyl- was second most abundant compound present in the essential oil. Furthermore, other prominent monoterpenes present in this essential oil were 3-Carene and 2-isopropyl-5-methylphenol.

**Table 1:** Thyme essential oil composition.

Peak	Run time	Area %	Name	Molecular formula	Molar mass (g/mol)	Classification	IUPAC name	Base m/z
1	3.440	45.58	1,3,8-p-Menthatriene	C_10_H_14_	134.22	Monoterpene	1-methyl-4-prop-1-en-2-ylcyclohexa-1,3-diene	119.15
2	3.831	12.63	3-Carene	C_10_H_16_	136.23	Monoterpene	3,7,7-trimethylbicyclo[4.1.0]hept-3-ene	93.05
3	5.580	0.10	3-Isopropylidene-5-methyl-hex-4-en-2-one	C_10_H_16_O	152.23	–	5-methyl-3-propan-2-ylidenehex-4-en-2-one	67.05
4	8.584	41.50	Phenol, 2-ethyl-4,5-dimethyl-	C_10_H_14_O	150.22	Phenol	2-ethyl-4,5-dimethylphenol	135.10
5	8.660	0.18	2-isopropyl-5-methylphenol	C_10_H_14_O	150.22	Monoterpene	5-methyl-2-propan-2-ylphenol	135.10

### Preparation of thyme oil nanoemulsion and its chitosan encapsulation

10% thyme oil was used invariably in preparing the oil in water nanoemulsion using high energy approach through ultrasonication. It was further encapsulated with chitosan and subjected to thermodynamic stability and centrifugation-based study for detecting any phase separation.

#### Thermodynamic stability

**Figure 2:**
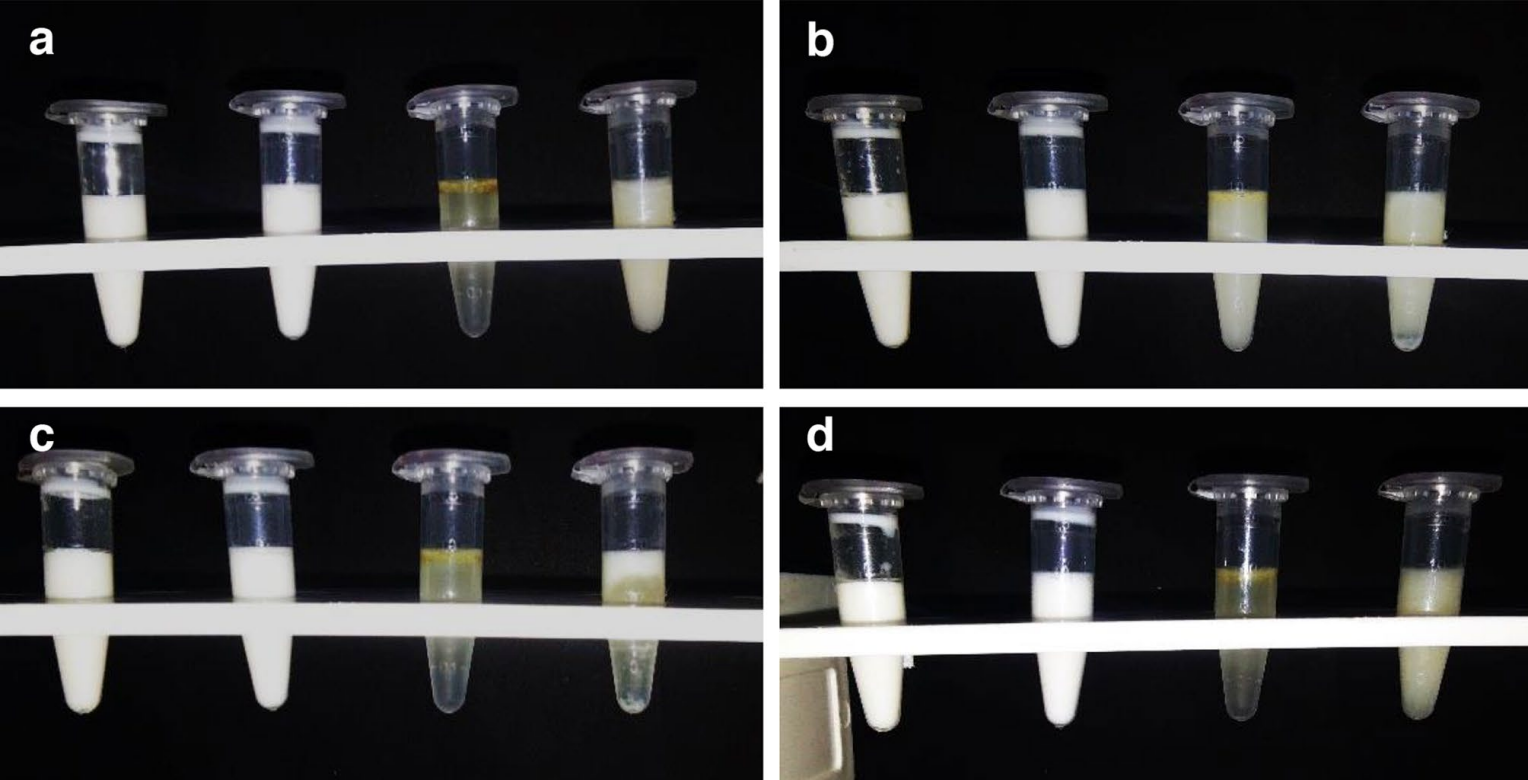
Stability analysis of thyme oil nanoemulsion (1:0.25, 1:0.5, 1:1 and 1:1.5) (**a**) Room temperature (**b**) cooling (**c**) centrifugation (**d**) heating.

**Figure 3:**
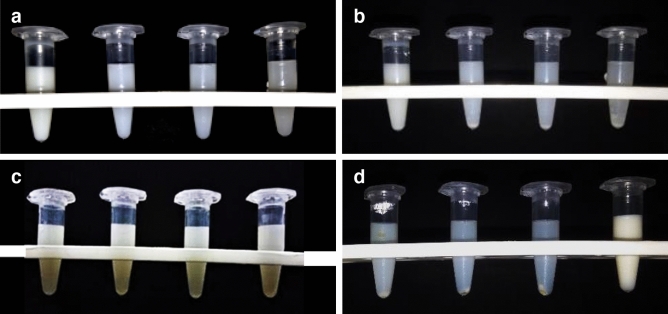
Stability analysis of chitosan encapsulation of thyme oil nanoemulsion (1:0.25, 1:0.5, 1:0.75 and 1:1) (**a**) Room temperature (**b**) cooling (**c**) centrifugation (**d**) heating.

It was seen that the nanoemulsions were stable at the room temperature for thyme oil, and 1:0.5 ratio (oil: surfactant) was the most stable after three months as shown in Fig. 2. At 4 °C the milky ness of all the ratios increased (Fig. 2b) while transparency increased after heating, in the thyme oil nanoemulsion (Fig. 2d). The stable thyme oil nanoemulsion ratio was further used for the preparation of chitosan encapsulation from which 1:1 ratio (thyme oil nanoemulsion: chitosan solution) was the most stable among all the varying ratios (Fig. 3) while slight precipitation was seen at the bottom of the Eppendorf tubes after cooling (Fig. 3b). A minute change in the texture of the nanoemulsion was observed after heating which could be seen as a slight increase in transparency of the nanoemulsions (Fig. 3d).

#### Centrifugation

Phase separation was observed in all the ratios for thyme oil nanoemulsion except in 1:0.5 (Fig. 2c) and for thyme oil nanoemulsion encapsulation a little amount of pellet was observed at the bottom of the tubes for all the ratios except 1:1 which was the most stable among all after three months.

#### pH study

**Figure 4:**
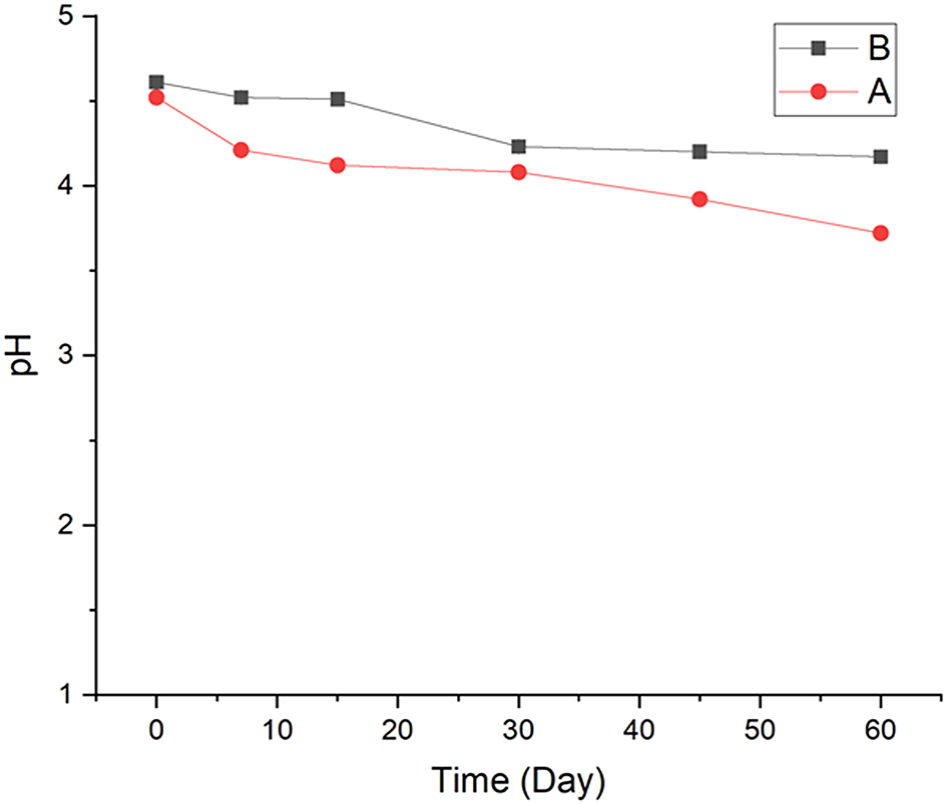
Alterations in pH of (A) thyme oil nanoemulsion and (B) its chitosan encapsulation over a period of two months.

pH of freshly prepared nanoformulations was measured and it was recorded as 4.52 ± 0.002 for thyme oil nanoemulsion and 4.61 ± 0.005 for its chitosan encapsulation which after two months, dropped upto 3.72 ± 0.007 and 4.17 ± 0.007 respectively. This slight alteration in acidic pH over time could be because of the presence of phenolic groups in the thyme oil. On comparing the both trends, there was a less drop in chitosan encapsulation which may be attributed to the higher stability imparted by the encapsulating material (Fig. 4).

### Characterization of thyme oil nanoemulsion and its chitosan encapsulation

**Figure 5:**
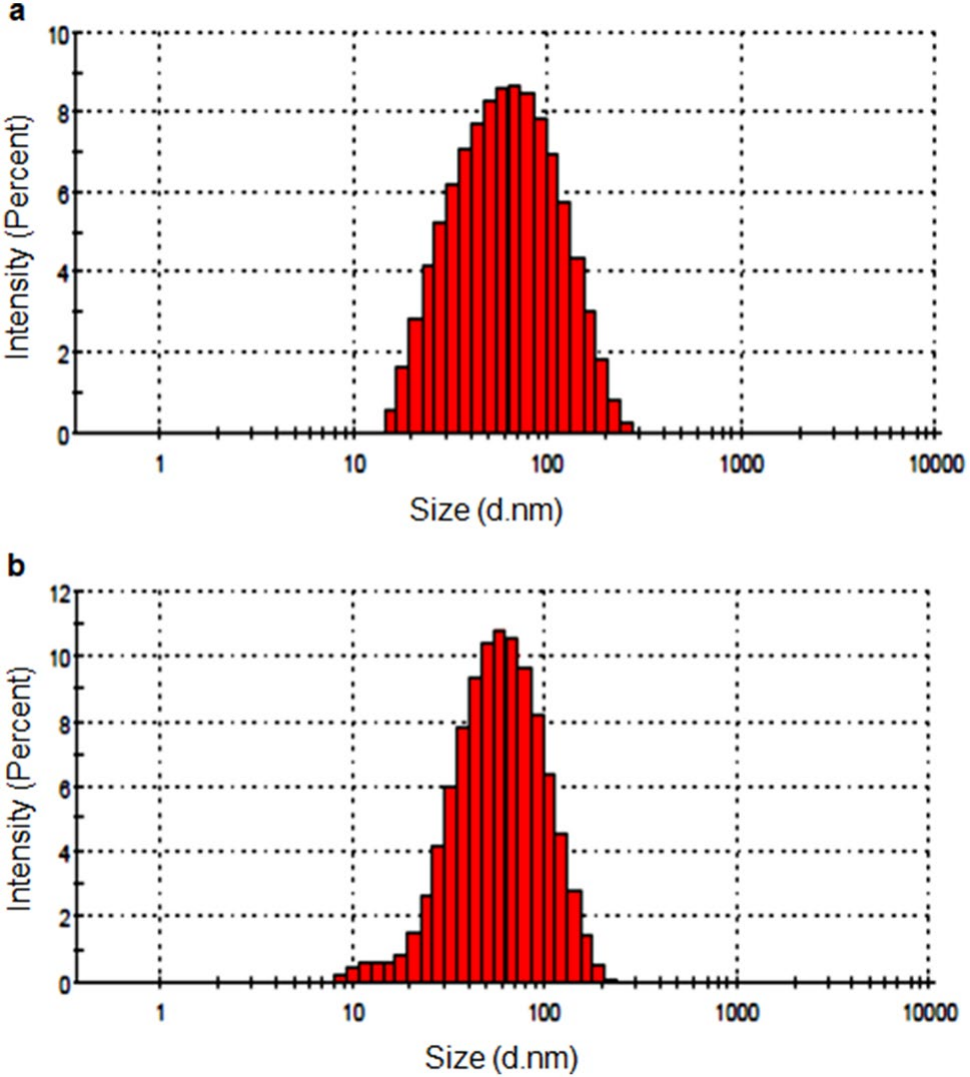
Size distribution by dynamic light scattering (**a**) thyme oil nanoemulsion (1:0.5) (**b**) chitosan encapsulation of thyme oil nanoemulsion (1:1).

Dynamic light scattering (DLS) is a technique used to measure colloidal stability through the measurement of the particle/droplet size and size distribution. DLS of thyme oil nanoemulsion and its chitosan encapsulation revealed the average hydrated size which was measured to be 52.18 ± 4.53 nm and 50.01 ± 2.32 nm respectively (Fig. 5). The PDI values were recorded as 0.237 ± 0.006 and 0.218 ± 0.005 respectively which showed that the formulations have the nano-size and the particles were monodispersed. Zeta potential observed for thyme oil nanoemulsion and its chitosan encapsulation was found to be 1.62 ± 0.052 mv and 0.733 ± 0.026 mv respectively.

**Figure 6:**
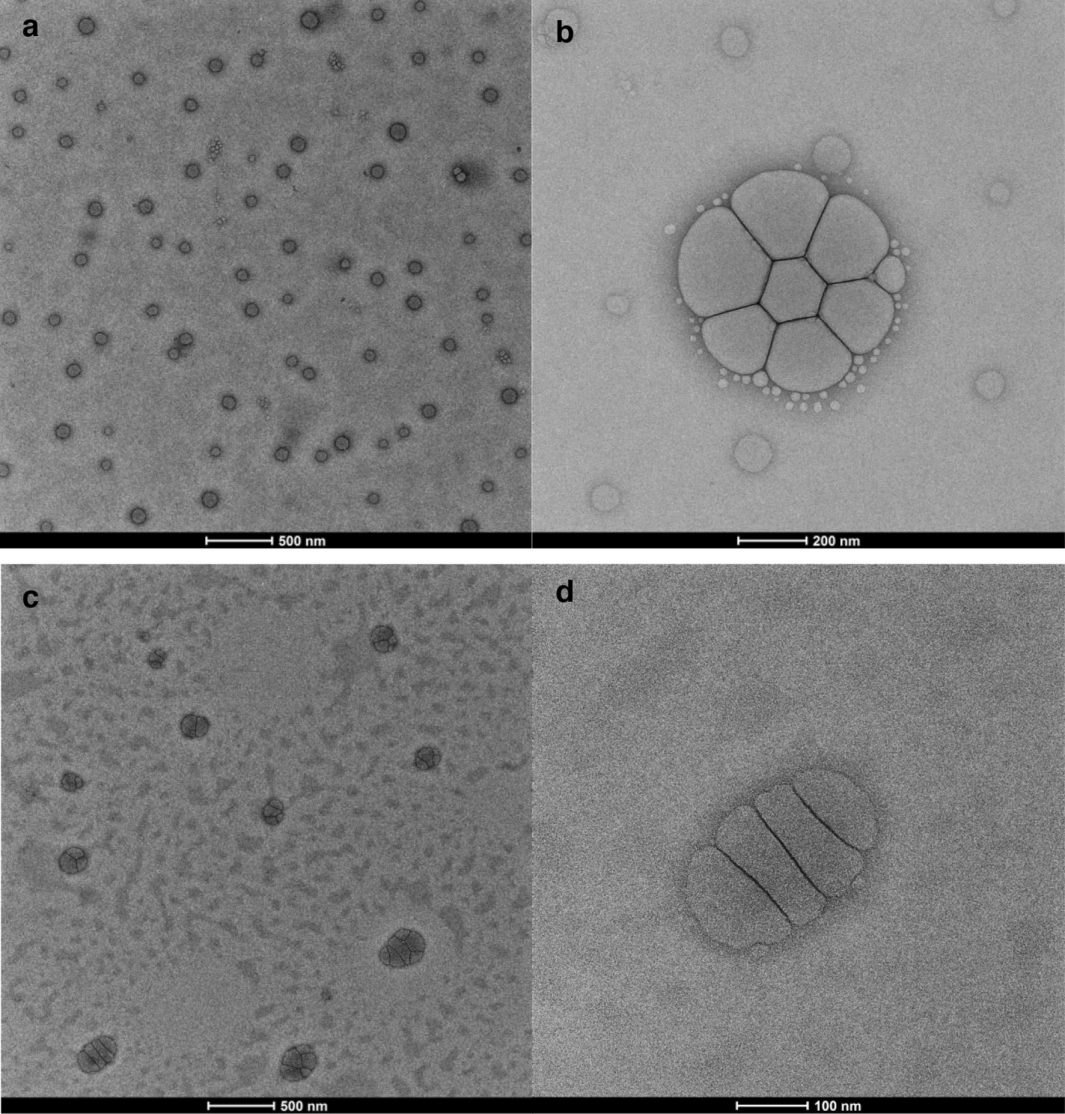
Transmission electron micrographs showing morphology of the nanoformulations (**a,b**) thyme oil nanoemulsion (**c,d**) chitosan encapsulation of thyme oil nanoemulsion.

Transmission electron microscopic analysis for thyme oil nanoemulsion exhibited that the size of droplets ranged from 40 to 110 nm which were spherical (Fig. 6a) however, these droplets tend to surround each other and acquire the shape of a distinct flower as evident from Fig. 6b. These flower-shaped droplets were uniformly dispersed with an average diameter of 684 nm where each petal measured 259 nm. This property was also noticed with the chitosan encapsulation of thyme oil nanoemulsion though slightly different shaped droplets could be noticed where each unit ranged between 76 and 126 nm as clearly depicted in Fig. 6c. A higher magnification electron micrograph (Fig. 6d) of encapsulation demonstrated that chitosan solution encapsulated the thyme oil nanoemulsion droplets which were uniformly clumped forming a distinct shape of mitochondria with droplets aligned in tandem. These shapes supplemented a unique characteristic to our nanoformulations.

### Release profile of thyme essential oil

**Figure 7:**
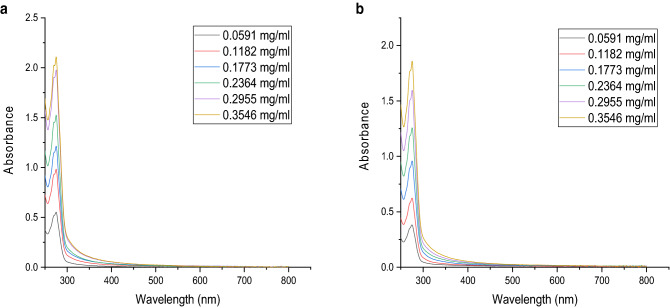
UV–Vis spectral peak of known concentration of (**a**) thyme oil nanoemulsion (**b**) chitosan encapsulation.

**Figure 8:**
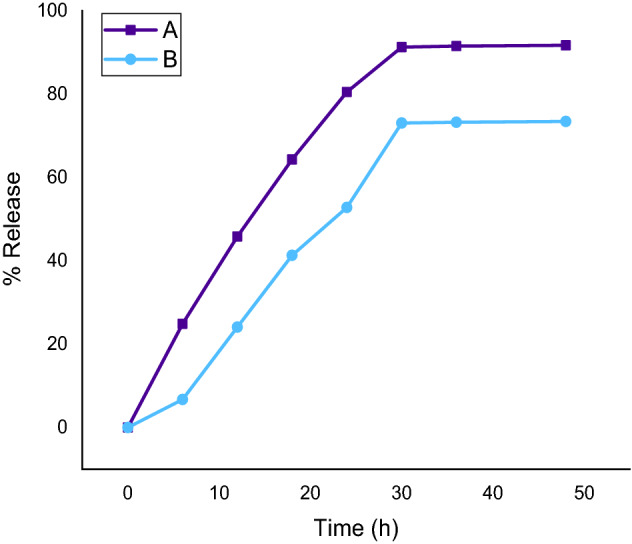
In-vitro release profile of thyme essential oil from (A) thyme oil nanoemulsion and (B) its chitosan encapsulation over a time period of 48 h.

The release profile of thyme essential oil from its nanoemulsion and chitosan encapsulation was studied using UV–Vis spectral analysis. Their distinct peaks were noticed at 275 nm and presented in Fig. 7 at a known concentration range (0.0591–0.3546 mg/ml). In-vitro release study of thyme essential oil from its nanoemulsion, showed that 91.68% of the total oil concentration in water was released while for its chitosan encapsulation the release of thyme essential oil was 73.41% after 48 h. There was a gradual decrease in the release of thyme essential oil from chitosan encapsulation as compared to nanoemulsion alone (Fig. 8) clearly depicting controlled release of thyme essential oil from its chitosan encapsulated nanoemulsion.

### Larvicidal bioassay

**Figure 9:**
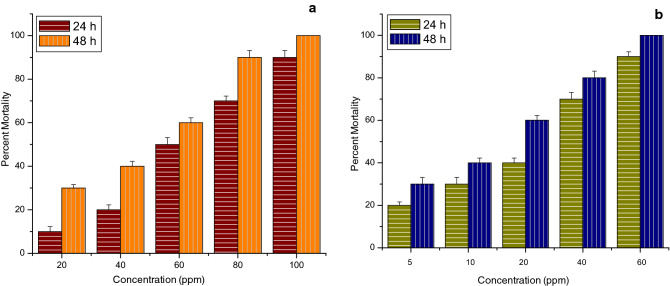
Graph depicting percent mortality of III instar larvae of *Anopheles stephensi* after 24 h and 48 h of exposure (**a**) thyme oil nanoemulsion (**b**) chitosan encapsulation of thyme oil nanoemulsion.

All the tests of the larvicidal activity of thyme oil nanoemulsion and its chitosan encapsulation were performed under laboratory conditions. The stable ratios of thyme oil nanoemulsion and its chitosan encapsulation were tested against III instar larvae of three mosquito species namely *Anopheles stephensi*, *A. aegypti* and *C. tritaeniorhynchus*. In the case of thyme oil nanoemulsion against *A. stephensi* highest percent mortality observed was 90% and 100% at 100 ppm after 24 h and 48 h respectively. While for its chitosan encapsulation it was observed at 60 ppm after the same time interval as represented in Fig. 9.

**Figure 10:**
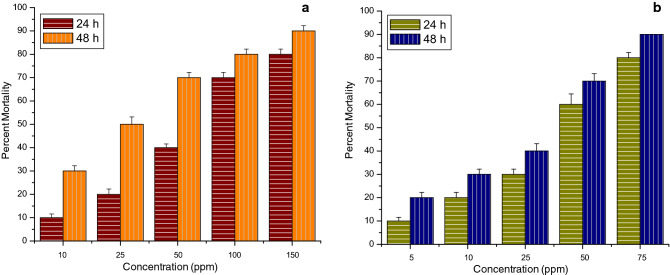
Graph depicting percent mortality of III instar larvae of *Aedes aegypti* after 24 h and 48 h of exposure (**a**) thyme oil nanoemulsion (**b**) chitosan encapsulation of thyme oil nanoemulsion.

Whereas, in the case of *A. aegypti* for thyme oil nanoemulsion the highest percent mortality observed was 80% and 90% at 150 ppm after 24 h and 48 h respectively. While for its chitosan encapsulation the same mortality was observed at only 75 ppm after the same time interval as shown in Fig. 10. This reveals that the chitosan encapsulation of thyme oil nanoemulsion is more effective.

**Figure 11:**
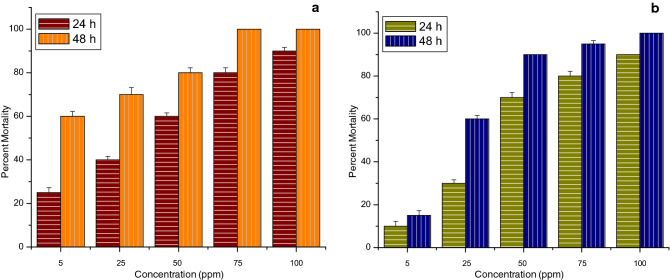
Graph depicting percent mortality of III instar larvae of *Culex tritaeniorhynchus* after 24 h and 48 h of exposure (**a**) thyme oil nanoemulsion (**b**) chitosan encapsulation of thyme oil nanoemulsion.

Moreover, in the case of *C. tritaeniorhynchus* not much difference was observed in the highest percent mortality of thyme oil nanoemulsion and its chitosan encapsulation as for thyme oil nanoemulsion 90% mortality was observed at 100 ppm after 24 h while 100% mortality was observed at 75 ppm after 48 h. further, for its chitosan encapsulation 90% and 100% mortality was observed at 100 ppm after 24 h and 48 h respectively. But it was observed that 25% and 60% mortality was observed at 5 ppm after 24 h and 48 h respectively. While 10% and 15% mortalities were observed at the same concentration and time interval as shown in Fig. 11. No mortality could be noticed in the negative control beakers and 100 percent mortality was observed with positive control of permethrin.

From the above data, the values for lethal concentration were calculated and represented in Tables 2 and 3. It is observed from Table 2 that after 24 h, LC_50_ value for *C. tritaeniorhynchus* was 22.58 ppm making this species to be most susceptible, followed by 56.13 ppm for *A. stephensi* and 58.72 ppm for *A. aegypti* concluding it to be least effective against *A. aegypti*. Similarly, it is observed from Table 3 that after 24 h LC_50_ value for *A. stephensi* was 18.88 ppm which is the least, then it is 29.8 ppm for *C. tritaeniorhynchus* and after that it is 33.63 ppm which is the highest for *A. aegypti* which shows that chitosan encapsulation of thyme oil nanoemulsion is most effective against *A. stephensi* and least effective on *A. aegypti*.

**Table 2:** Larvicidal activity of thyme oil nanoemulsion against III instar larvae of *Anopheles stephensi*, *Aedes aegypti* and *Culex tritaeniorhynchus* after 24 h and 48 h.

Mosquito sp.	Time (h)	Equation	LC_50_ (ppm) (LFL-UFL)	LC_90_ (ppm) (LFL-UFL)	chi square
*A. stephensi*	24	y = 3.708x − 1.487	56.13 (46.2–67.35)	124.38 (96.2–206.5)	2.96
	48	y = 3.158x + 0.028	37.49 (27.89–46.11)	95.44 (73.6–155.5)	7.29
*A. aegypti*	24	y = 1.933x + 1.581	58.72 (41.74–85.44)	270.33 (126.53–510.67)	0.728
	48	y = 1.48x + 2.97	23.49 (11.86–35.86)	172.5 (96.38–652.01)	0.179
*C. tritaeniorhynchus*	24	y = 1.375x + 3.138	22.58 (11.72–35.32)	193.13 (100.88–849.46)	3.593
	48	y = 1.191x + 4.227	4.45 (0.59–9.54)	53.0 (28.13–183.55)	6.76

*LFL* Lower fiducial limit, *UFL* Upper fiducial limit.

**Table 3:** Larvicidal activity of chitosan encapsulation of thyme oil nanoemulsion against III instar larvae of *Anopheles stephensi*, *Aedes aegypti* and *Culex tritaeniorhynchus* after 24 h and 48 h.

Mosquito sp.	Time (h)	Equation	LC_50_ (ppm) (LFL-UFL)	LC_90_ (ppm) (LFL-UFL)	chi square
*A. stephensi*	24	y = 1.824x + 2.67	18.88 (12.98–27.21)	95.23 (55.48–299.5)	2.51
	48	y = 1.97x + 2.887	11.82 (7.65–16.37)	52.87 (34.19–123.8)	3.39
*A. aegypti*	24	y = 1.737x + 2.35	33.63 (23.29–53.2)	183.8 (97.3–729.2)	1.81
	48	y = 1.617x + 2.839	21.66 (14.18–32.65)	134.28 (72.96–493.5)	3.07
*C. tritaeniorhynchus*	24	y = 2.039x + 1.99	29.8 (19.63–41.13)	126.79 (83.35–278.9)	2.59
	48	y = 2.409x + 2.125	15.59 (9.92–21.67)	53.08 (37.7–87.84)	1.5

*LFL* Lower fiducial limit, *UFL* Upper fiducial limit.

### Morphological analysis

**Figure 12:**
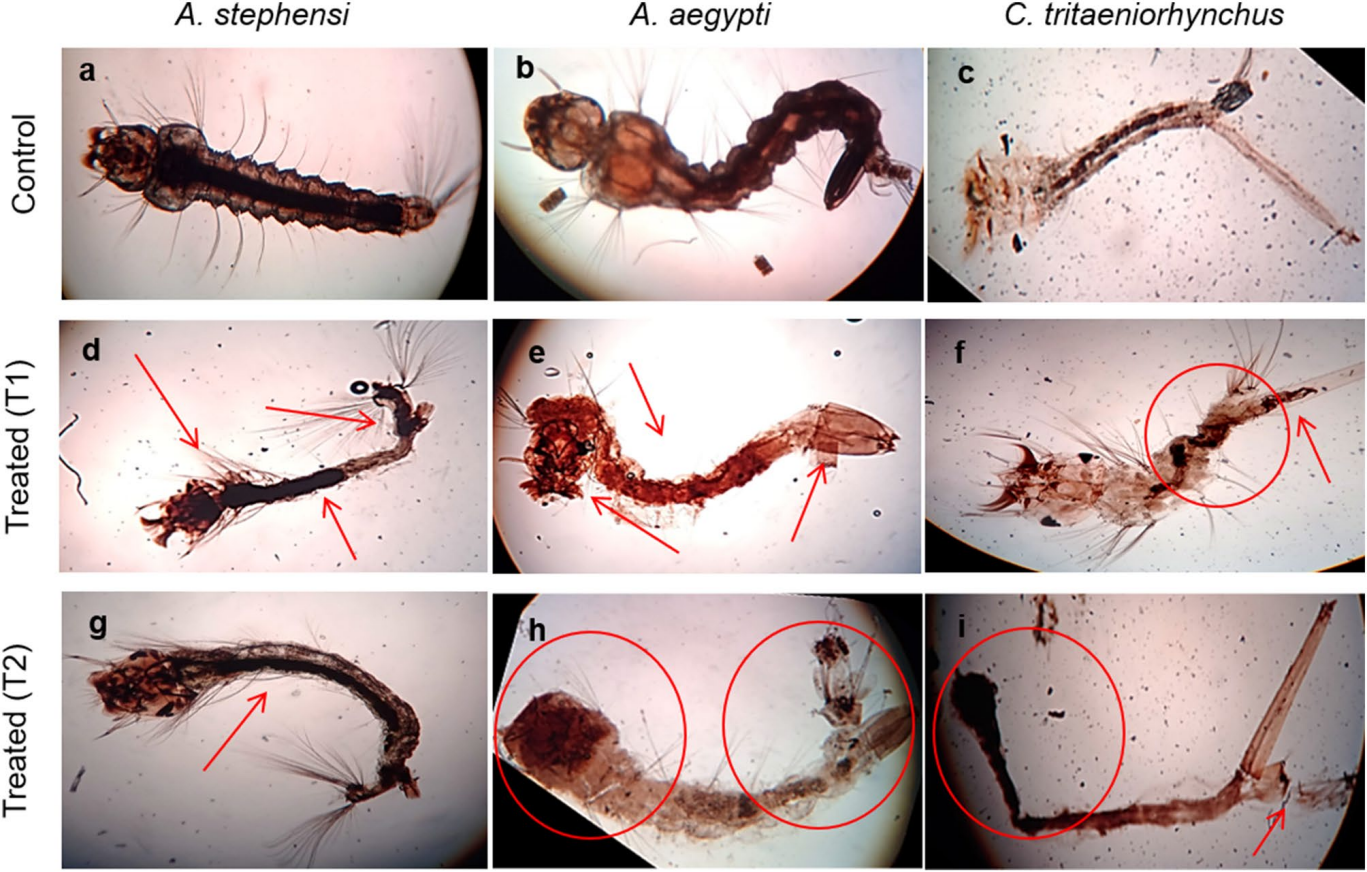
Comparative depiction of alterations in the morphology of various III instar larvae following exposure with thyme oil nanoemulsion (T1) and chitosan encapsulation of thyme oil nanoemulsion (T2).

The effect of prepared nanoformulations was also observed on the morphology of treated larvae which were noticed to change from the standard control morphology as shown in Fig. 12. In the present scenario, as a result of the application of thyme oil nanoemulsion and its chitosan encapsulation, gross morphological changes also occurred in the *A. stephensi*, *A. aegypti* and *C. tritaeniorhynchus* larvae. A clear change was visible in whole segments of the body i.e. the control is seen with well-developed and elaborate segments while in the treated larva, the segments could not be exclusively identified as separate fragments. The body also became constricted and distorted towards the anal region abruptly. The bristles on the setae were reduced and could not be identified as they were in the control specimen. There was a general blackening and shrinking of the body with distorted internal body parts in the treated larva.

## Discussion

Mosquitoes can be considered as the undisputed kings of disease transmission having vectorial capabilities responsible for some of the most deadly and feared diseases of the world. Previously, to curb the menace caused by the mosquitoes, several artificially synthesized chemical insecticides like malathion and permethrin were used but with time, the vectors developed a resistance to these chemicals. Another negative impact could be seen as harmful effects on humans and other animals. More acute toxicity of permethrin was noticed in the children as compared to adults. Due to all these unwanted effects, plant-based and natural herbal products need to be favored as they are safer. The important products currently under focus are the essential oils obtained from various plants. Essential oils are an important natural resource for obtaining insecticides.

In our study, 1,3,8-p-Menthatriene (45.58%) was the highest reported phyto-compound from the thyme essential oil. Moreover, phenolic compound- Phenol, 2-ethyl-4,5-dimethyl- (41.50%) was also present. The activities of the thyme oil are attributed to the monoterpenes and phenolic phyto-compounds which are similar with the previous findings^26,27^.

In the present study 1:0.5 (oil: surfactant) ratio of thyme oil nanoemulsion was found to be the most stable at room temperature as evident from extensive thermodynamic stability analysis and further validated through centrifugation and pH measurements. Moreover, there was no flocculation or phase separation observed, this may be because of the small size which was further confirmed by DLS and TEM. This stable ratio was further used for the chitosan encapsulation, among the four prepared ratios of thyme oil nanoemulsion to chitosan 1:1 ratio was found to be the most stable after the thermodynamical, centrifugal test and pH which were further analyzed by DLS and TEM. According to DLS the size was 52.01 ± 4.53 nm and 50.18 ± 2.32 nm for thyme oil nanoemulsion and its chitosan encapsulation respectively with the PDI value 0.237 ± 0.006 and 0.218 ± 0.005, the size is comparatively smaller than the nanoemulsion synthesized from mandarin essential oil (z-diameter 176.4 nm and PDI 0.22)^28^. The change in the size of nanoemulsion is due to the use of different surfactant and method of preparation as the size depends on the intermixing. They employed Tween 20 surfactant and used homogenization method for preparing the nanoemulsion which resulted in larger droplet size. However, in our study the use of Tween 80 and high energy ultra sonication approach might have led smaller droplet size^23^.

A comparative study of thyme oil nanoemulsion and its chitosan encapsulation was conducted for evaluating larvicidal effect against *A. stephensi*, *A. aegypti* and *C. tritaeniorhynchus*. The nanoemulsion of the oil was encapsulated with chitosan for controlled release and utilized surfactant Tween 80 for better lipophilicity^29^.

When seen in the terms of percent mortality of test organisms, it can be inferred that the encapsulated thyme oil nanoemulsion was more active, faster acting and potent against *A. stephensi* and *A. aegypti* whereas thyme oil nanoemulsion was more effective against *C. tritaeniorhynchus*. In the present study, chitosan encapsulated thyme oil nanoemulsion was far more effective in case of *A. stephensi* and *A. aegypti* since 90% mortality in the former case is attained at 60 ppm as compared to the 100 ppm in the latter case when observed at 24 h. Chitosan being a natural, biodegradable encapsulating material, probably helps in the bioadhesion with the outer skin of the mosquito larvae and provides the better activity. Similarly, 100% mortality was achieved in the former at 60 ppm as compared to the 250 ppm in the latter when observed at 48 h. Other essential oils have also been checked for their larvicidal efficiency after being encapsulated. *Piper aduncum* and *Piper hispidinervum* essential oils were encapsulated in gelatin nanoparticles and larvae of *A. aegypti* were exposed to these nanoparticles which gave 100% mortality at 500 ppm after 24 h^30^ while the thyme oil nanoemulsion and its chitosan encapsulation were more potent presenting that chitosan is an extremely superior encapsulating material than gelatin. Effectiveness of encapsulated thyme oil nanoemulsion may be attributed to the controlled release of thyme essential oil. In previous studies also the encapsulated nanoemulsion exhibited potent activities due to the controlled release^14,30^.

The LC_50_ values of thyme oil nanoemulsion and chitosan encapsulated thyme oil nanoemulsion were 56.13 ppm and 18.88 ppm against *A. stephensi*, 58.72 ppm and 33.63 ppm against *A. aegypti* and 22.58 ppm and 29.8 ppm against *C. tritaeniorhynchus* after 24 h respectively, which are in accordance with the results obtained in other experiments^31^ using only thyme oil solution on *A. stephensi*, where the LC_50_ ranged from 9.7 to 101.4 ppm after 24 h. In an extensive review work done, it was observed that the LC_50_ values of essential oils of different *Thymus* plants ranged from 23 to 43 ppm for larvae of different species of *Culex*^32^ while in the present study the LC_50_ values were less which implies increased potency and efficiency of the thyme essential oil upon being subjected to nano formulation and encapsulation.

The larvicidal activities of the essential oil of *Nigella sativa* after 24 h of exposure period were reported to be higher (LC_50_ 53.9 ppm, LC_90_ 172.6 ppm) for *A. stephensi*^33^ and while those for thyme oil nanoemulsion (LC_50_—18.88 ppm and LC_90_—95.23 ppm) and its encapsulation (LC_50_—56.13 ppm and LC_90_—124.38 ppm) remarkably lower lethal concentrations were obtained in the present study for the same test organism and same time period. The χ^2^ value thyme oil nanoemulsion and its encapsulation showed a significant decrease of 2.96 and 2.51 from the 9.2 of the essential oil of *N. sativa*.

The LC_50_ values of various essential oils of different plants like 11.36 ppm for nanoemulsion of Tarragon essential oil against *A. stephensi*^34^, 38.8 ppm for nanoemulsion of Dill essential oil against *A. stephensi*^35^, 17.76, 17.5, 25.9, 20.6 ppm for *Trachyspermum ammi, Smyrnium olusatrum*, *Pimpinella anisum*, *Helosciadium nodiflorum* respectively against *Cx. quinquefasciatus*^36^ while our study showed that the thyme oil nanoemulsion and its chitosan encapsulation have very low LC_50_ and LC_90_ values against all the selected species which proved them as a better alternative to many of the previously tested essential oil emulsions or nanoemulsions.

The differences observed in the percent mortality and lethal concentrations values mainly depend on the characteristic of the nanoemulsion formed. In the present study, due to the controlled release, the chitosan encapsulated nanoemulsion showed better activity as compared to thyme oil nanoemulsion which was also reported in previous study^14^. TEM studies revealed the size in the range between 40 and 110 nm for thyme oil nanoemulsion and 76.4 nm (individual) for its chitosan encapsulation which was comparatively smaller when compared with the size of chitosan loaded nanoemulsion containing *Zataria multiflora* (130.20 nm) and *Bunium persicum* (154.26 nm) essential oils^37^. In another study, the TEM revealed the size of 9.4 nm for eucalyptus nanoemulsion^38^ which is very small as compared to our study. However, the unique flower-shaped droplets for thyme oil nanoemulsion and mitochondrion shape for its encapsulation were observed and reported here for the first time. The smaller size gives the larger surface area for the drug reaction and absorption also the unique bonding found in our study gives special binding properties to the nanoformulations which enhanced the larval efficacies.

Gross morphological alterations were also noticed in the III instars larvae of all the treated species. All the segments of the larval body were deformed as the cuticles get damaged following treatment with thyme nanoemulsion and nanoencapsulation. Moreover, excessive shrinkage was also demonstrated in these larvae. This study of morphological analysis is in accordance with previous researchers^39,40^ who also reported the damage in external structure and shrunken cuticles in *A. aegypti* due to the herbal extract. These cuticular deformations in anal region and body led to the interruption in ion regulation and osmotic changes which probably resulted in the mortality.

A work similar to the present work has been done with essential oil of *Siparuna guianensis* was encapsulated in chitosan nanoparticles and tested for prolonged mosquitocidal activity against the larvae of *A. aegypti*. It was seen that all chitosan: essential oil ratios evaluated had better larvicidal activity than just the pure oil without adjuvants which may be attributed to the characteristic flower shape and considerably smaller droplet size of synthesized nanoemulsions in this study. On the other hand, the higher the proportion of essential oil in the nanoparticle, the higher was the larvicidal activity observed^41^. Recently, it was reported that thymol-eugenol nanoemulsion was effective against *A. aegypti* at a very low concentration^42^. All these parameters and results hold true for the present experimental solution of thyme oil nanoemulsion and its chitosan encapsulation too.

## Methods

### Materials

The essential oil of thyme used in the preparation of nanoemulsion was procured from the authorized dealer. Tween 80 (Polyoxyethylene 80 sorbitan monooleate) was purchased from Merck and used as a surfactant for nanoemulsion preparation while for encapsulation, 100% deacetylated chitosan was obtained from Sigma Aldrich.

### Collection and maintenance of mosquito larvae

Larvae of *A. stephensi*, *A. aegypti* and *C. tritaeniorhynchus* were collected separately from different local sites of Agra region (27.176° N and 78.008° E) and maintained under the standard laboratory conditions at a temperature of 28 ± 2 °C in the distilled water and were acclimatized. They were provided with Brewer’s yeast tablets as a food for larvae and the water was changed on alternate days. Further, the III instar larvae were used for the larvicidal bioassay.

### Gas chromatography and mass spectrometry (GC–MS)

Thyme oil has been screened for the identification of bioactive components by using GC–MS triple quadrupole (GC–MS TQ8030, Shimadzu Corp., Japan). Gas chromatography attached with Restek column (0.25 mm, 30 m, Rxi-5 ms) which was operated using Q3 scan acquisition mode (start time 3 min, end time 10 min, scan speed 2500, start m/z 40 and end m/z 700). The ionization voltage was 70 eV. The sample was introduced via all-glass injector working in the split mode and helium was used as a carrier gas (flow rate of 1 ml/min keeping the split ratio of 10:1). Initially, column temperature was kept at 100 °C, then increased linearly at 3 °C/min up to 300 °C and held for 5 min. The temperature of injection port was 250 °C and the GC–MS/MS interface was maintained at 300 °C. The identification of components was accomplished by comparing retention time and fragmentation pattern, as well as with mass spectra in the NIST spectral library stored in the computer software of the GC–MS/MS^43^.

### Preparation of thyme oil nanoemulsion

The nanoemulsion of *Thymus vulgaris* oil was formulated with a slight modification in a method given by Sugumar et al.^38^. The mixture of thyme oil and non-ionic surfactant (Tween 80) was stirred on the magnetic stirrer and milli-Q water was added intermittently. The concentration of Thyme oil was fixed for all the formulations. Four ratios (oil: surfactant) were synthesized (1:0.25, 1:0.5, 1:1 and 1:1.5). Oil in Water nanoemulsion (v/v) was prepared by adding surfactant, oil and milli-Q water by subjecting them to ultrasonication (performed for 15 min at 25% amplitude with 10–03 s pulse). All the prepared ratios were subjected to stability analysis and the most stable ratio was used for larvicidal bioassay.

### Preparation of encapsulated thyme oil nanoemulsion

For preparing encapsulation, initially, the solution of 100% deacetylated chitosan was prepared by the method given by Jamil et al.^44^ with minor modification. 0.3% chitosan solution was prepared in 1% of glacial acetic acid. Concomitantly, 0.1% of emulsifying agent, sodium tripolyphosphate (STPP) was also prepared. Now, the 0.3% solution of chitosan (100 ml, pH- 5.0) and 0.1% solution of STPP (4 ml, pH- 9.0) were mixed together, the pH value was adjusted to 5.0 and the mixture was continuously stirred at 60 °C and 700 rpm for 6 h. After this, well-amalgamated mixture was subjected to bath sonication for 30 min and later on centrifuged at 25 °C at 8000 rpm for 15 min. The pellet was discarded after the centrifugation and the supernatant was collected and stored. Thereafter, the stable ratio of oil-in-water thyme nanoemulsion was mixed with chitosan solution in four different ratios namely 1:0.25; 1:0.5; 1:0.75 and 1:1, which were again subjected to sonication (for 15 min at 25% amplitude with 10–03 s pulse). Afterwards, the prepared chitosan encapsulation of thyme oil nanoemulsion was subjected to stability analysis and characterization.

### Characterization studies

#### Thermodynamic stability

The stability of the nanoformulations was checked at low temperature (4 °C), room temperature (25 °C ± 2 °C) and under heat stress (50–55 °C).

#### Centrifugation

The formulated nanoemulsions were centrifuged at 3000 rpm for 10 min and were observed for phase separation, if any.

#### pH

The pH values of the nanoformulations were determined directly using a calibrated digital pH meter (Mettler Toledo, FiveEasy, FE20-I, Switzerland) at room temperature. The analyses were performed in triplicates.

#### Dynamic light scattering (DLS)

The droplet size and Polydispersity index (PDI) of thyme oil nanoemulsion was determined using a Malvern Zeta Sizer. Nanoemulsions were diluted with water (1:4) to reduce multiple scattering effects before each experiment. The Polydispersity index (PDI) was used to characterize the distribution of size.

#### Transmission electron microscopy (TEM)

This was done for visualizing the shape and morphology of the synthesized nanoemulsions and its encapsulation. The drop of nanoemulsion was negatively stained with phosphotungstic acid and was placed on a copper grid. The images were obtained using a transmission electron microscope (Talos 200 kV TEM) at AIIMS, New Delhi.

### Release profile of thyme essential oil

The release study of thyme oil from nanoformulations was performed by using UV–Vis spectrophotometric method using Eppendorf BioSpectrometer (India)^45^. However, major modifications were made in this method briefly described here. Firstly, the spectral scanning was performed over a range from 250 to 800 nm with chitosan solution (0.3%), thyme oil nanoemulsion (1:0.5 ratio) and its chitosan encapsulation at various concentration range. As a distinct peak was noticed at the wavelength of 275 nm, hence, absorbance for a range of known concentrations (0.0591, 0.1182, 0.1773, 0.2364, 0.2955, 0.3546 mg/ml selected after screening of a wide range of concentrations to get a detectable spectral peak) was recorded at this wavelength for plotting the standard curve for thyme oil nanoemulsion and its encapsulation. For in vitro release profiling, the absorbance was noted at the time interval of every 6 h for a period of 48 h at the highest concentration (0.3546 mg/ml). The gradual change in the absorbance was noticed and the concentrations were calculated with the help of standard curve however, the percent release of oil in the air was calculated as follows: (TCW − CW) × 100/TCW. Where, TCW = Total concentration of oil in water and CW = Concentration of oil in water after specific time interval.

### Larvicidal bioassay

The larvicidal bioassay was performed as per the standard methods recommended by the World Health Organization^46^ with minor modification. Synthesized nanoformulation toxicity test was performed by introducing twenty III instar mosquito larvae of *A. stephensi*, *C. tritaeniorhynchus* and *A. aegypti* in each of the 250 mL sterile beaker containing 100 mL of water. After adding larvae to beakers, a range of increasing concentrations of the stable ratio of thyme oil nanoemulsion and its encapsulation were added to each of the beakers, separately. Each test was carried out in five replicates and an equal number of controls were set up and run along with the test set up. Negative control (water) and positive control (permethrin, concentration: 1 ppm) were set up. The temperature was 28 °C ± 2 °C and the larvae were provided with the photoperiod of 12 h D : 12 h L (Dark: Light). No food was given during the time of bioassay. The larvicidal effects of the nanoformulations were monitored by recording mortality after 24 h and 48 h of the exposure period for determining toxicity against III instar larvae of *A. stephensi*, *A. aegypti* and *C. tritaeniorhynchus*.

### Statistical analysis

All the data of average larval mortality were subjected to Probit analysis by which the lethal concentration (LC_50_ and LC_90_) values, other statistics at 95% fiducial limits of upper confidence limit and lower confidence limit, and chi-square values were calculated using Finney’s table^47^. If the negative control mortality was observed to fall in between 5% and 20%, the mortalities of treated groups were corrected according to Abbott’s formula^48^, Abbott’s formula = [(T − C)/(100 − C)] × 100, Where, T = percentage mortality of test organism; C = percentage mortality of control.

### Morphological analysis

After performing the bioassay, the control and treated larvae were stored separately in the 70% alcohol. Later, they were subjected to dehydration by passing them through an increasing dehydration series viz*.* 80%, 90% and 100%. Afterward, the larvae were permanently mounted with DPX on glass slides, pictures were taken under the light microscope under × 4 magnification and the pictures of treated larvae were compared with the pictures of control larvae for observing the morphological variations.

## Conclusions

From the present study, it may be concluded that the thyme oil nanoemulsion and its chitosan encapsulation possess larvicidal efficacy which can establish them as very competent and efficient agent against the mosquito vectors. The thyme oil nanoemulsion (1:0.5) and its chitosan encapsulation (1:1) were found to be stable till three months and were potent against III instar larvae of *A. stephensi*, *A. aegypti* and *C. tritaeniorhynchus*. Also, the geometry found in TEM images were unique flower shaped.

Moreover, chitosan encapsulated nanoemulsion showed controlled release of thyme essential oil and was potent herbal mosquito larvicide as the lethal concentrations are low. Further work awaits where the refinement of the encapsulated particles will be performed and also the test of this encapsulated nanoemulsion and thyme oil nanoemulsion against many other pests and vectors is done not only through aqueous medium but also with several other methods of bioassay.
